# Non-radiative origin for alpine endemics of *Draba* (Brassicaceae) in the central mountains of the Japanese Archipelago

**DOI:** 10.1007/s10265-025-01643-7

**Published:** 2025-04-29

**Authors:** Ryutaro Koda, Yoshinori Murai, Hajime Ikeda

**Affiliations:** 1 Department of Multidisciplinary Sciences, Graduate School of Arts and Sciences, The University of Tokyo, 3-8-1 Komaba, Meguro-ku, Tokyo 153-8902 Japan; 2 Department of Botany, National Museum of Nature and Science, 4-1-1 Amakubo, Tsukuba, Ibaraki 305-0005 Japan

**Keywords:** Alpine plants, Beringia, BioGeoBEARS, Biogeography, East-Central Asia, Phylogeography

## Abstract

Diversity of endemic species may result from two evolutionary processes: *in-situ* diversification (radiation) or phylogenetically independent divergences (non-radiation). To explore the evolutionary history of endemic-rich alpine flora in the Japanese Archipelago, we investigated the phylogenetic relationships of four endemic taxa of *Draba* (Brassicaceae) from the high mountains of central Honshu: *D. kitadakensis*, *D. sachalinensis* var. *shinanomontana*, *D. sakuraii* var. *nipponica*, and *D. shiroumana*. Using molecular phylogenetic analyses on plastid *trnL-F* and nuclear ITS sequence of 157 taxa, we found that the four taxa endemic to central Honshu did not form a monophyletic group and diverged from at least two evolutionary independent lineages. Moreover, ancestral area reconstruction further revealed that some of their ancestral species may have originated from different geographical regions. These findings indicate that the endemic *Draba* in central Honshu diverged through non-radiative evolutionary origin. Our study suggests that the richness of endemic species in the alpine zone of the Japanese Archipelago is associated with multiple sources with high species diversity located in nearby geographical regions.

**Supplementary Information:**

The online version contains supplementary material available at 10.1007/s10265-025-01643-7.

## Introduction

Cold-adapted plants inhabiting high mountains, such as alpine plants, are renowned for their exceptionally high diversity of endemic species (Hughes and Atchison 2015). One scenario explaining this high diversity of endemic species is in situ diversification (radiation) (Rahbek et al. 2019; Rangel et al. 2018), a commonly observed pattern across various mountain ranges (e.g., Madagascar, Camacho et al. 2021; European Alps, Comes and Kadereit 2003; Rocky Mountains, Drummond et al. 2012; the Andes, Hughes and Eastwood 2006). This pattern of evolutionary history is largely driven by the “island-like” geographically isolated distribution of alpine zones (Hughes and Eastwood 2006), which limits the gene flow (Morgan and Venn 2017), and enhances genetic differentiation (DeChaine and Martin 2005). In addition, complex topography in mountain regions provides mosaic environmental conditions, which lead to local adaptation and subsequent divergence of endemic species (DeChaine et al. 2014). Thus, endemic species in a particular mountain range will form monophyly under this evolutionary history (Hughes and Eastwood 2006). On the other hand, endemic species may diverge from phylogenetically independent lineages (non-radiation). In this case, each species originated through migrations—including long-distance dispersal—from multiple source areas and/or independent adaptation events, resulting in polyphyletic relationships (e.g., Dagallier et al. 2020; Salomón et al. 2022; Smyčka et al. 2022). Since the evolutionary history of endemic species diversity was limited to studies in major mountain ranges (e.g. the Andes, Rocky Mountains, and European Alps; see Hughes and Atchison 2015 for a comprehensive review), it remains to be explored which of these evolutionary histories could explain the high endemism in mountain ranges with limited geographic extension.

The high mountains of the Japanese Archipelago host species-rich alpine flora, of which approximately 51.3% of its alpine flora is endemic to the archipelago, making it a significant hotspot of endemic species (Kato and Ebihara 2011; Shimizu 1982, 1983). Given that nearly half of alpine endemics to the archipelago have closely related species distributed in the Arctic, northern Pacific (Beringia), northern Asia, and North America (Shimizu 1983), it implies that the ancestor of endemic species likely derived from cold-adapted plants distributed in these regions. Since the Japanese Archipelago had minimum glacial coverage during the glacial periods of the Pleistocene climatic oscillation (Tsukada 1983), cold-adapted plants could have migrated to the archipelago during these periods as far as central Honshu, the main island of the Japanese Archipelago (Ikeda 2022; but see Ikeda et al. 2014 for exception), and accumulated unique genetic divergence in numerous alpine plants (Fujii and Senni 2006; Ikeda 2022). Moreover, the alpine zone of central Honshu harbors several genera containing multiple endemic species, for example, *Leontopodium* (Pers.) R.Br. ex Cass., *Taraxacum* F. H. Wigg., and *Ranunculus* L. (Kato and Ebihara 2011; Shimizu 1982, 1983). However, the evolutionary history of high endemic species diversity on high mountains in the Japanese Archipelago has never been explored.

*Draba* Dill. ex L. (Brassicaceae) is one of the most species-rich genera (ca. 390 species) that occur throughout the major mountain systems and arctic tundra around the world (Jordon-Thaden and Koch 2008; Jordon-Thaden et al. 2010, 2013). *Draba* provide an appropriate study system to explore the evolutionary origin of the high endemic species diversity in mountain flora. Although some species are widely distributed in circum-polar areas and temperate mountains, the majority of species are endemic to narrow geographical regions (Jordon-Thaden et al. 2013), representing a significant proportion of total species richness in the mountainous flora (Figueroa et al. 2022; Jordon-Thaden et al. 2013). Regardless of the high diversity of endemic species in this genus, a previous study investigated phylogenetic relationships among ca. 43% of 169 species in *Draba* using both plastid (*trnL-F*) and nuclear (ITS) DNA sequences (Jordon-Thaden et al. 2010), providing a framework for further phylogenetic comparisons (Kucs et al. 2021).

**Fig. 1:**
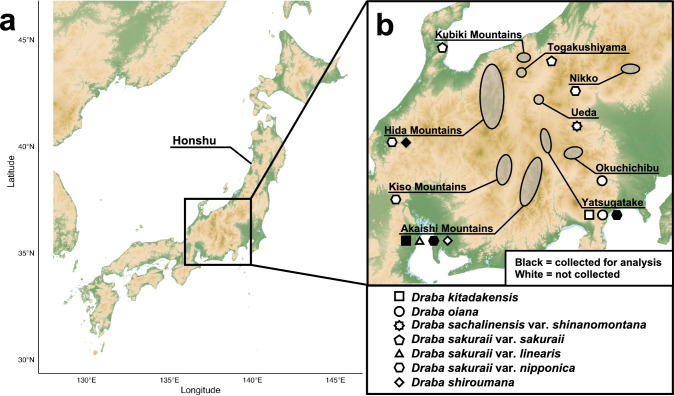
Topological map of **a** the Japanese Archipelago and **b** distribution of endemic *Draba* in central Honshu. Black and white shapes indicate whether samples were collected for the present study or not

In the alpine zone of the Japanese Archipelago, *Draba* is the most endemic species-rich genus containing11 endemic taxa (Kadota 2016; Kato and Ebihara 2011) of which seven occur in the mountains of central Honshu (Kadota 2016): *Draba kitadakensis* Koidz. found in the Akaishi Mountains and Yatsugatake; *Draba oiana* Honda, restricted to Okuchichibu and Yatsugatake; *Draba sachalinensis* (F. Schmidt) *Trautv.* var. *shinanomontana* (Ohwi) Okuyama occurring only in Ueda, Nagano Prefecture; *Draba sakuraii* Makino var. *sakuraii*, confined to the Kubiki Mountains and Togakushiyama; *Draba sakuraii* var. *linearis* (Kitam.) Sugim., found exclusively in the Akaishi Mountains; *Draba sakuraii* var. *nipponica* (Makino) Takeda, sporadically occurring across Nikko, the Hida Mountains, Kiso Mountains, Akaishi Mountains, and Yatsugatake; *Draba shiroumana* Makino, endemic to the Hida and Akaishi Mountains (Fig. 1; Kadota 2016). Of these seven endemic taxa, only *D. sachalinensis* var. *shinanomontana* has been analyzed in the previous phylogenetic study (accession B161 corresponding to the herbarium voucher B100127351, Curator Herbarium B 2000 in Jordon-Thaden et al. 2010). Among the remaining taxa, three varieties of *D. sakuraii* differ only in their hair density (Kadota 2016), while *D. oiana* is distinguished from *D. kitadakensis* only in the number of stem leaves and style length (Kadota 2016), and is often considered as synonym with *D. kitadakensis* (Kato and Ebihara 2011; Ohwi 1965). According to this classification, the phylogenetic relationships among the three robust species, *D. sakuraii*, *D. kitadakensis*, and *D. shiroumana*, may inform about the evolutionary history of the endemic *Draba* to central Honshu.

In this study, we aim to clarify the evolutionary history that led to the high diversity of endemic species in the alpine zone of the Japanese Archipelago, through unraveling the phylogenetic relationship of the endemic taxa of *Draba*. We newly sequence the plastid (*trnL-F*) and nuclear (ITS) DNA of the three species *D. kitadakensis*, *D. sakuraii* var. *nipponica*, and *D. shiroumana*, and investigate their phylogenetic relationships with other *Draba species* covered in the previous study (Jordon-Thaden et al. 2010). Furthermore, we reconstruct the ancestral area for each endemic taxa to infer their phylogeographical origins. Through these analyses, we examine whether the endemic *Draba* to central Honshu have a radiative or non-radiative origin. If these endemic taxa have a radiative origin, a monophyletic relationship among these endemic species is expected. Alternatively, if these endemic taxa have a non-radiative origin, they would present polyphyletic relationships, each being closely related to species outside the Japanese Archipelago.

## Materials and methods

### Chloroplast and nuclear DNA sequencing

Leaf material of three endemic taxa of *Draba* were collected from the following sites: *D. kitadakensis* from Kitadake (Akaishi Mountains), *D. sakuraii* var. *nipponica* from Yatsugatake and Kitadake, and *D. shiroumana* from Shiroumadake (Hida Mountains) (Table 1). DNA of two samples of each endemic taxon was extracted from a silica gel dried sample using DNeasy Plant Mini Kit (Qiagen, Hilden, Germany). The intron and intergenic spacer of chloroplast and nuclear regions were amplified and sequenced. For the chloroplast DNA, the *trnL–F* region was obtained through the separate amplification of the *trnL* introns and the *trnL–F* intergenic spacer using the following primers: *trnL* intron forward primer 5′-CGA AAT CGG TAG ACG CTA CG-3′; *trnL* intron reverse primer 5′-GGG GAT AGA GGG ACT TGA AC-3′; intergenic spacer forward primer 5′-CGA AAT CGG TAG ACG CTA CG-3′; intergenic spacer reverse primer 5′-ATT GAA CTG GTG ACA CGA G-3′ (Taberlet et al. 1991). For the nuclear DNA, the internal transcribed spacer (ITS) region of nuclear ribosomal DNA including ITS1, 5.8S, and ITS2 were amplified using the ITS-18 forward primer 5′-GCA TGT TTT CCC AGT CAC GAC GGA AGG AGA AGT CGT AAC AAG G-3′ and the ITS-25 reverse primer 5′-ACT TCA GGA AAG AGC TAT GAC GGG TAA TCC CGC CTG ACC TGG-3′. The PCR amplifications were performed using fast reaction polymerase (SapphireAmp® Fast PCR Master Mix; TaKaRa BioInc., Shiga, Japan), following the manufacturer’s protocol, in which thermal cycling condition involved initial denaturation at 94 °C for 1 min, followed by 30 cycles at 98 °C for 5 s, 55 °C for 5 s, 72 °C for 10 s, and a final extension at 72 °C for 5 min. These amplicons were purified using ExoSAP-IT (USB Corporation, Ohio, USA) and used as direct sequencing templates. Cycle sequencing was conducted with a BigDye Terminator 3.1 cycle sequencing kit (Applied Biosystems, Foster City, California, USA), and the reaction mixture was sequenced using an ABI 3130 Genetic Analyzer (POP-7 polymer and 50-cm capillary; Applied Biosystems, Foster City, CA).

**Table 1:** Plant materials collected in this study

Taxon and locality	Latitude	Longitude	Sample ID
*Draba kitadakensis* Koidz			
Kitadake, Akaishi Mountains, Yamanashi Prefecture	35.70	138.2	7968
Kitadake, Akaishi Mountains, Yamanashi Prefecture	35.70	138.2	7969
*Draba sakuraii* Makino var. *nipponica* Makino			
Yatsugatake Mountains, Nagano Prefecture	35.98	138.37	6258
Kitadake, Akaishi Mountains, Yamanashi Prefecture	35.68	138.24	7522
*Draba shiroumana* Makino			
Shiroumadake, Hida Mountains, Nagano Prefecture	36.79	137.79	7966
Shiroumadake, Hida Mountains, Nagano Prefecture	36.79	137.79	7967

### Phylogenetic analysis and haplotype network

Chromatograms of obtained sequenced data were manually checked and the contig was created using ChromasPro 2.1.10.1 and combined with published *trnL–F* and ITS sequences used in Jordon-Thaden et al. (2010). In order to reduce the bias in subsequent phylogenetic analysis, we retained only the core *Draba* (as defined in Jordon-Thaden et al. 2010) and *Pseudoturris turrita* (L.) Al-Shehbaz, a sister taxa to *Draba* (Jordon-Thaden et al. 2010)*,* as the outgroup. To account for the polyphyletic nature of several species, potentially due to the existence of cryptic species (Jordon-Thaden et al. 2010), we treated each sample as a distinct molecular lineage, in which each lineage was identified by its species epithet and internal sample ID (see Table S1 in Supplementary Information). Subsequently, all the sequences were aligned using MUSCLE (Edgar 2004), built in AliView (Larsson 2014).

For phylogenetic analyses, each sequence dataset was analyzed by maximum likelihood (ML) using RAxML-NG 1.2.0 (Kozlov et al. 2019) and Bayesian Inference (BI) using BEAST 2 2.7.6 (Bouckaert et al. 2014). MEGA11 (Tamura et al. 2021) was used to select the best-fitting substitution model for each DNA region, and the best-fit model was chosen based on the Akaike information criterion (AIC) (Akaike 1974). For ML analysis, the bootstrap supports (BS) were assessed using 1,000 bootstrap pseudo-replicates. For BI analysis, four simultaneous Monte Carlo Markov chains (MCMCs) were run for 1.0 × 10^8^ generations, and trees were sampled every 1,000 generations. The first 10% were discarded as burn-in.

For the haplotype network, a subset of sequence data was created based on the phylogenetic tree constructed in the previous step. Samples belonging to a clade included all endemic species in central Honshu and with strong support (either BS > 80 or PP > 0.95) were used. The network was constructed using the TCS network method conforming to POPART (Leigh and Bryant 2015).

### Ancestral area reconstruction

To reconstruct the ancestral distribution of endemic *Draba* taxa to central Honshu, we defined seven geographical regions: (A) Eurasian Arctic, encompassing Arctic Asia, Lappland, coastal Norway, and the North Atlantic Islands; (B) Eurasian Beringia, encompassing Chukchi/Kamchatka, Wrangel Island, Cherskii/Kolyma Mountain/Tundra, and the Kuril Islands; (C) East-Central Asia, encompassing of the Greater Asian Mountains, along with the Korean Peninsula and Okhotsk; (D) Japanese Archipelago; (E) Western Eurasia, encompassing the Mediterranean, Central Europe, Turkey/Caucasus/Iran, and Atlantic European forests/grasslands; (F) North America, encompassing North American Beringia, the North American Cordillera, Eastern North America, and Greenland; and (G) South America. This classification basically depends on ecoregions that explain species composition of *Draba* (Jordon-Thaden et al. 2013), whereas we designated the Japanese Archipelago as an independent geographic area. This classification aligns with defining Beringia as extending from west of the Lena River to east of the Mackenzie River (Abbott and Brochmann 2003), thereby excluding both the Korean Peninsula and Okhotsk. To prevent overestimation of the distribution range for species containing potential cryptic species, each lineage was assigned a single geographical region described above based on the geographical coordinates of each sample provided by Jordon-Thaden et al. (2010). The best consensus tree topology and 1,000 randomly sampled trees of BI phylogeny from prior analyses were then used to examine a likelihood-based biographical model in BioGeoBears (Matzke 2013) implemented in RASP 4.3 (Yu et al. 2020). The model test for BioGeoBears was conducted using three different models: DEC, DIVALIKE, and BAYAREALIKE (Matzke 2013). Despite the ongoing controversy over the jump dispersal (+ J) model (Matzke 2014, 2022; Ree and Sanmartín 2018), we also tested this model, considering the island-like nature of the alpine zone (DeChaine and Martin 2005). The best-fit model was chosen based on the corrected AIC (AICc).

## Results

### Phylogenetic analysis

The genus-wide *trnL-F* dataset contained 351 samples comprising 157 taxa, and their sequences consisted of a total of 907 bp with 299 variable sites and 175 parsimony informative sites. Besides, the ITS dataset contained 352 samples comprising 157 taxa, and their sequences consisted of a total of 625 bp with 265 variable sites and 175 parsimony informative sites. The best-fit substitution model was selected as GTR + G + I for both *trnL-F* and ITS regions. The phylogenetic trees constructed using *trnL–F* showed low interspecific polymorphism, while trees based on ITS (Figs. S1, S2) showed greater interspecific variation. Since many branches displaying near-zero lengths in *trnL–F* (Figs. S3, S4) unlikely reflect divergent history among species, we solely used ITS trees for further analyses.

**Fig. 2:**
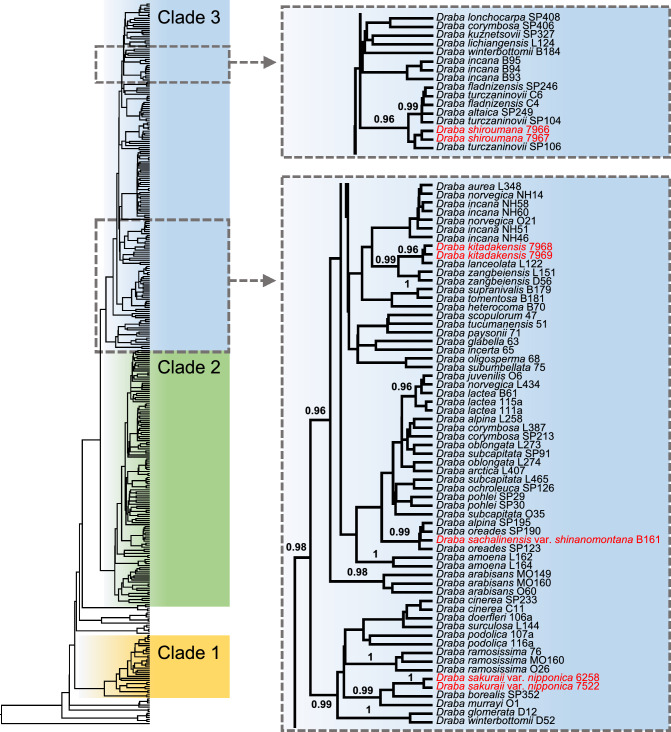
Bayesian tree based on ITS. Three highly supported clades (PP ≥ 0.95) are highlighted. Two topologies, detailing the relationships of endemic taxa from central Honshu and their closely related taxa, are shown. Endemic taxa from central Honshu are indicated with colored text. Posterior probability support values (PP ≥ 0.95) are shown

The BI tree constructed from the ITS supported three distinct clades with high posterior probabilities (PPs > 0.95) (Fig. 2). Clade 1 primarily comprised taxa distributed across Western Eurasia, while Clade 2 was dominated by taxa from East-Central Asia, North America, and South America. Clade 3 included taxa predominantly from the Eurasian Arctic, Eurasian Beringia, East-Central Asia, and North America. All endemic taxa from central Honshu belonged to Clade 3 (Fig. 2). While two samples of each *D. kitadakensis*, *D. sakuraii* var. *nipponica*, and *D. shiroumana* formed monophyly per taxa, and there was not a monophyletic relationship among the four endemic species (Fig. 2). In particular, two clades strongly supported a polyphyletic relationship, one clade containing *D. sakuraii* var. *nipponica* (PP = 0.99) and another clade containing *D. kitadakensis*, *D. sachalinensis* var. *shinanomontana*, and *D. shiroumana* (PP = 0.96). The sister lineage for each endemic taxon was estimated as follows: *D. kitadakensis* with *D. lanceolata* Royle (L122; central China; PP = 0.96), *D. sachalinensis* var. *shinanomontana* with *D. oreades* Schrenk (SP190; the Altay Mountains) + *D. alpina* (SP195; the Polar Urals) (PP = 0.99), *D. sakuraii* var. *nipponica* with *D. borealis* D.C. (SP352; Kunashir Island in the Kuril Islands; PP = 0.86); *D. shiroumana* with *D. turczaninowii* Pohle & N. Busch (SP106; East Sayan; PP = 0.57) (Fig. 2).

### Haplotype network

**Fig. 3:**
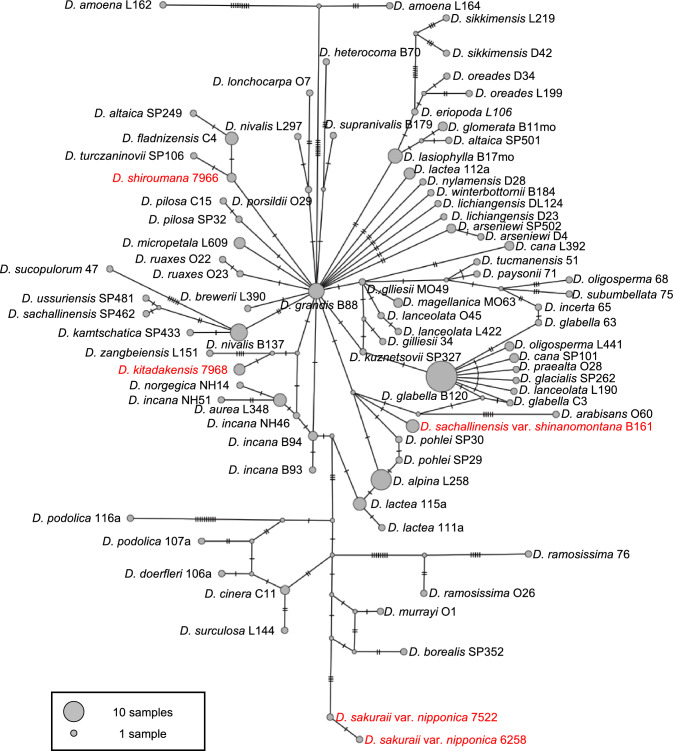
Haplotype network of *Draba* belonging to clade 3 from the Bayesian tree based on ITS. Each tick on the line represents a nucleotide polymorphism, and the circumference of each circle is proportional to the number of taxa with no nucleotide polymorphism. Endemic taxa from central Honshu are indicated with colored text

The haplotype network was constructed with samples belonging to clade 3 in the phylogenetic tree (Figs. 2, 3). Among the three endemic taxa that included more than one sample, *D. sakuraii* var. *nipponica* was the only polymorphic, while others (*D. kitadakensis* and *D. shiroumana*) had no nucleotide polymorphisms (Fig. 3). In congruence with the relationships observed in the phylogenetic tree (Fig. 2), *D. kitadakensis* was found to share a haplotype with *D. lanceolata* (L122), *D. sachalinensis* var. *shinanomontana* shared a haplotype with *D. oreades* Schrenk (SP123; SP190) and *D. alpina* (SP195), while *D. sakuraii* var. *nipponica* differed from *D. borealis* (SP352) by 5 to 6 nucleotides, and *D. shiroumana* differed from *D. turczaninowii* (SP106) by one nucleotide (Fig. 3).

### Reconstruction of ancestral area

**Fig. 4:**
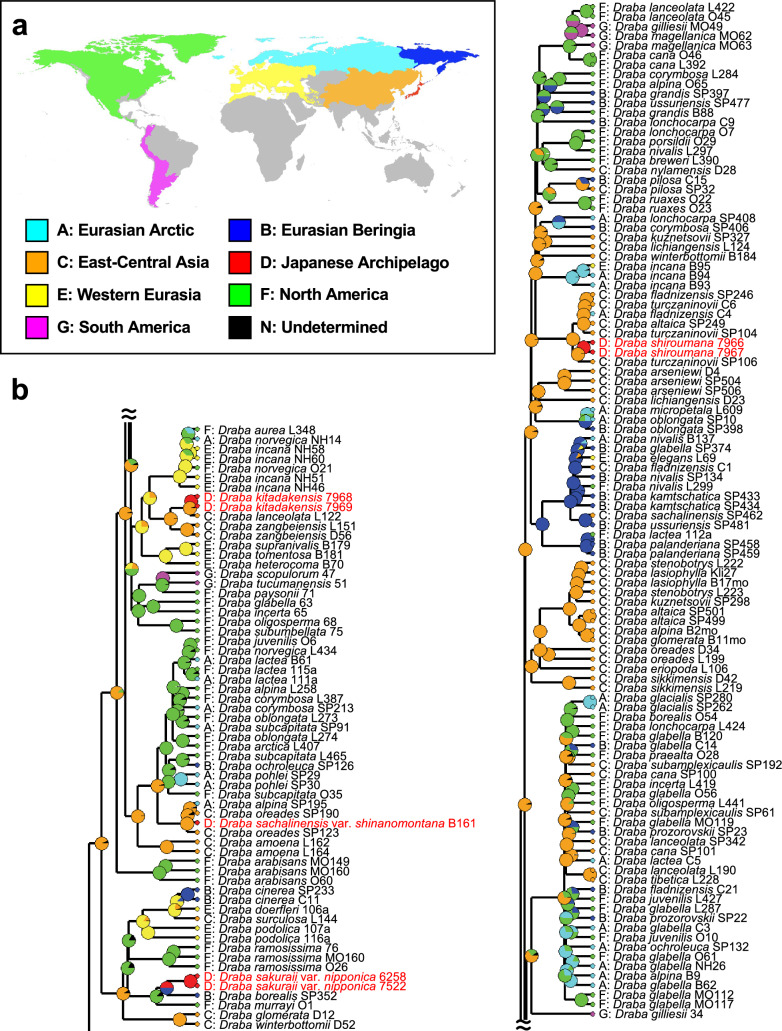
Ancestral area reconstruction of the *Draba* belonged to clade 3 from the Bayesian tree based on ITS. **a** Distribution map of the seven geographical regions used for reconstruction. **b** Estimated ancestral areas and likelihoods inferred using BioGeoBEARS under best-fitting model BAYAREALIKE + J. The alphabet beside taxonomic name represents the geographical region used for reconstruction. Endemic taxa from central Honshu are indicated with colored text

Ancestral area reconstruction was performed using the best-fitting model, such as the BAYAREALIKE + J model (Table 2). Ancestral area reconstruction estimated that clade 1 originated from Western Eurasia (E) (ancestral area likelihood [AAL] = 100.0%; Fig. S5), and clades 2 and 3 originated from East-Central Asia (C) (AAL = 99.7% and 92.3%, respectively; Figs. 4, S5). The ancestral area(s) of the endemic taxa from central Honshu was reconstructed as the following: *D. kitadakensis* and its sister lineage, *D. lanceolata* (L122), was East-Central Asia (C) (AAL = 94.8%); *D. sachalinensis* var. *shinanomontana* and its sister lineage, *D. oreades* (SP190) and *D. alpina* (SP195), was East-Central Asia (C) (AAL = 90.8%); *D. sakuraii* var. *nipponica* and its sister lineage, *D. borealis* (SP352), was Eurasian Beringia (B) + Japanese Archipelago (D) (AAL = 50.4% and 49.6%, respectively); and *D. shiroumana* and its sister lineage, *D. turczaninowii* (SP106), was East-Central Asia (C) (AAL = 96.9%) (Fig. 4).

**Table 2:** Log-likelihood (LnL) and corrected Akaike information criterion (AICc) of the six different models tested using BioGeoBEARS

Models	LnL	AICc
BAYAREALIKE + J	− 411.6	829.3
DEC + J	− 411.7	829.5
DIVALIKE + J	− 412.6	831.2
DEC	− 483	969.2
DIVALIKE	− 494.1	992.2
BAYAREALIKE	− 508.6	1021

## Discussion

The present study revealed the four endemic taxa of *Draba* to high mountains in central Honshu—*D. kitadakensis*, *D. sachalinensis* var. *shinanomontana*, *D. sakuraii* var. *nipponica*, and *D. shiroumana*—are not monophyletic (Fig. 2). Even though the phylogenetic trees have little resolution for relationships among closely related species due to generally low branch supports (< 0.95), *D. sakuraii* var. *nipponica* and the remaining three taxa (*D. kitadakensis*, *D. sachalinensis* var. *shinanomontana*, and *D. shiroumana*) belonged to different clades with high support values (PP = 0.99 and 0.96, respectively) (Figs. 2, S2). This finding implies that endemic taxa of *Draba* in central Honshu originated from at least two different evolutionary lineages. Consequently, our study suggests that the evolutionary history associated with richness of the endemic *Draba* in central Honshu is compatible with the idea of a non-radiative origin, instead of diversifying from a single common ancestor as expected in cases denoting a radiative origin (Hughes and Eastwood 2006).

We acknowledge several cautions in our phylogenetic analyses due to the low resolution in molecular data (Jordon-Thaden et al. 2010; Kucs et al. 2021). In particular, polyphyletic relationships may represent the lack of phylogenetic information in the sequence data due to random sorting of ancestral polymorphisms during rapid speciation. However, the phylogenetic relationships mostly exhibited geographic patterns (Fig. 4), which unlikely resulted from random sorting of polymorphisms (Goodwillie and Stiller 2001; Ikeda 2022). Thus, the independent lineage of *D. sakuraii* var. *nipponica* from the remaining species would represent a robust evolutionary history. On the contrary, the low resolution of our phylogenetic trees prevents us from leading to the non-radiative origin of *D. kitadakensis*, *D. sachalinensis* var. *shinanomontana*, and *D. shiroumana*, even though they grouped with different species (Fig. 4).

In addition, it should be noted that the present study did not analyze all taxa endemic to central Honshu. Given the morphological similarity of *D. sakuraii* var. *linearis* and *D. sakuraii* var. *sakuraii* to *D. sakuraii* var. *nipponica* and *D. oiana* to *D. kitadakensis* (Kadota 2016; Kato and Ebihara 2011), the unanalyzed species may be closely related to the species analyzed in this study. Although some of their diversification may reflect a radiative origin, their radiative origin could not exclude the present finding that endemic *Draba* originated from at least two independent lineages.

Considering the evolutionary history noted in the non-radiative origin of alpine endemics to major mountain ranges (e.g., Dagallier et al. 2020; Salomón et al. 2022; Smyčka et al. 2022), the high endemism of *Draba* in the Japanese Archipelago could be explained by a similar scenario to the divergence of *Helichrysum* Mill. (Asteraceae) in the Drakensberg, the southernmost alpine zone in Africa (Blanco-Gavaldà et al. 2023). In *Helichrysum*, 12 species are endemic to the Drakensberg, which belong to at least seven distinct lineages. In particular, four lineages originated from nearby species-rich regions, three from the southern African grasslands (which harbor 98 species) and the one from the Fynbos biome (which contains 12 species) (Blanco-Gavaldà et al. 2023). This divergent history implies that multiple nearby regions with high species diversity may be associated with local high endemism, where migration of distantly related lineages from multiple sources contributes to endemic species with non-radiative origin within the alpine zone (Dagallier et al. 2020). In the case of the endemic *Draba*, the Japanese Archipelago is located nearby areas with high species diversity such as the Eurasian Arctic, Eurasian Beringia, and East-Central Asia, with 40, 43, and 99 species, respectively (Jordon-Thaden et al. 2013). The present polyphyletic relationships among the endemic taxa of *Draba* (Fig. 2), and different geographical origins estimated as their ancestral area (Fig. 4) suggest migrations from multiple regions, implying non-radiative origin of the high endemism of *Draba* in the Japanese Archipelago. Given that almost half of the endemic species in the alpine zone in the Japanese Archipelago are related to species distributed in the Arctic, northern Pacific, and northern Asia (Shimizu 1983), multiple endemic species in other alpine plant genera, such as *Leontopodium*, *Taraxacum*, and *Ranunculus,* may have non-radiative origin from these species-rich regions.

## Conclusion

We showed that endemic taxa of *Draba* to central Honshu originated from at least two evolutionary independent lineages, suggesting that their divergence occurred in a non-radiative manner. Our finding in the alpine zone of the Japanese Archipelago did not align with the radiative origin of endemic species diversity commonly observed in several major mountain ranges (Hughes and Atchison 2015). In contrast, the present non-radiative origin is explained by a similar scenario with the previous studies (Blanco-Gavaldà et al. 2023; Dagallier et al. 2020); multiple source regions with high species diversity led to the high endemism of *Draba* in the Japanese Archipelago. 

## Supplementary Information

Below is the link to the electronic supplementary material.

Supplementary file1 (PDF 1501 KB)

Supplementary file2 (XLSX 39 KB)

## Data Availability

All sequence data generated and analyzed publicly available in the NCBI database. Accession numbers and related details are provided in Supplementary file 2.
