# Supplementary material for: Non-radiative origin for alpine endemics of Draba (Brassicaceae) in the central mountains of the Japanese Archipelago
Source: J Plant Res. 2025 Apr 29;138(4):563–73. doi: 10.1007/s10265-025-01643-7 (PMC12238178; doi:10.1007/s10265-025-01643-7)
Supplement: Supplementary file 1 — Supplementary file1 (PDF 1501 KB) [file 10265_2025_1643_MOESM1_ESM.pdf]

**Electronic supplementary materials**

**Title:** Non-radiative origin for alpine endemics of *Draba* (Brassicaceae) in the central mountains of the Japanese Archipelago

**Journal:** Journal of Plant Research

**Authors:** Ryutaro Koda[1], Yoshinori Murai[2], Hajime Ikeda[1]

**Affiliation:**

[1] Department of Multidisciplinary Sciences, Graduate School of Arts and Sciences, The University of Tokyo, 3-8-1 Komaba, Meguro-ku, Tokyo 153-8902, JAPAN.

[2] Department of Botany, National Museum of Nature and Science, 4–1–1 Amakubo, Tsukuba, Ibaraki, 305–0005 Japan

**Email Address:** koda-ryutaro-herb@g.ecc.u-tokyo.ac.jp

**Content:**

Fig. S1 The maximum likelihood tree based on ITS. The asterisk indicates strong bootstrap support (BS >= 80), and BS values for Japanese endemic taxa and their neighboring taxa are shown. The sample IDs are provided after the taxonomic name. Endemic taxa from central Honshu are indicated in colored text.

Fig. S2 The Bayesian tree based on ITS. The asterisk indicates strong posterior probability support (PP >= 95), and PP values for Japanese endemic taxa and their neighboring taxa are shown. The sample IDs are provided after the taxonomic name. Endemic taxa from central Honshu are indicated in colored text.

Fig. S3 The maximum likelihood tree based on *trnL-F*. The asterisk indicates strong bootstrap support (BS >= 80), and BS values for Japanese endemic taxa and their neighboring taxa are shown. The sample IDs are provided after the taxonomic name. Endemic taxa from central Honshu are indicated in colored text.

Fig. S4 The Bayesian tree based on *trnL-F*. The asterisk indicates strong posterior probability support (PP >= 95), and PP values for Japanese endemic taxa and their neighboring taxa are shown. The sample IDs are provided after the taxonomic name. Endemic taxa from central Honshu are indicated in colored text.

Fig. S5. Ancestral area reconstruction of the genus *Draba*. (a) Distribution map of the seven geographical regions used for reconstruction. (b) Bayesian tree based on ITS with estimated ancestral areas and likelihoods inferred using BioGeoBEARS, represented as pie charts at each node. Endemic taxa from central Honshu are indicated in colored text. Posterior probability support (PP ≥ 95) and PP values for neighboring taxa of the endemic *Draba* from central Honshu are indicated.

Table S1. List of plant materials used in this study, including internal accession ID for each sample, geographical area IDs for BioGeoBEARS analysis, GenBank accession numbers for sequences used in the phylogenetic analyses and latitude and longitude of collection sites. All sequences of samples not described as newly collected in this study were published in Jordon-Thaden et al. (2010).

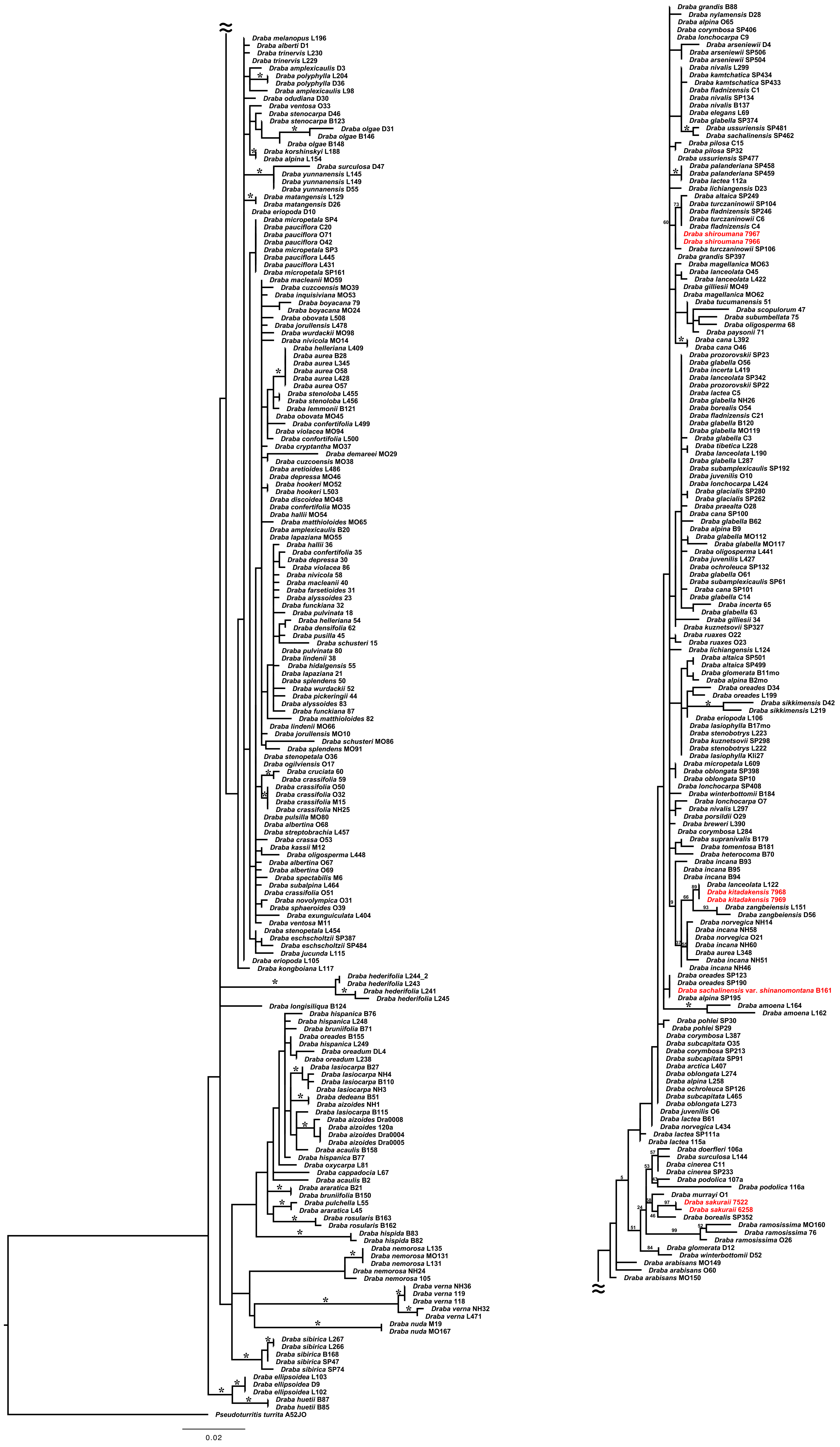


Fig. S1 The maximum likelihood tree based on ITS. The asterisk indicates strong bootstrap support (BS >= 80), and BS values for Japanese endemic taxa and their neighboring taxa are shown. The sample IDs are provided after the taxonomic name. Endemic taxa from central Honshu are indicated in colored text.

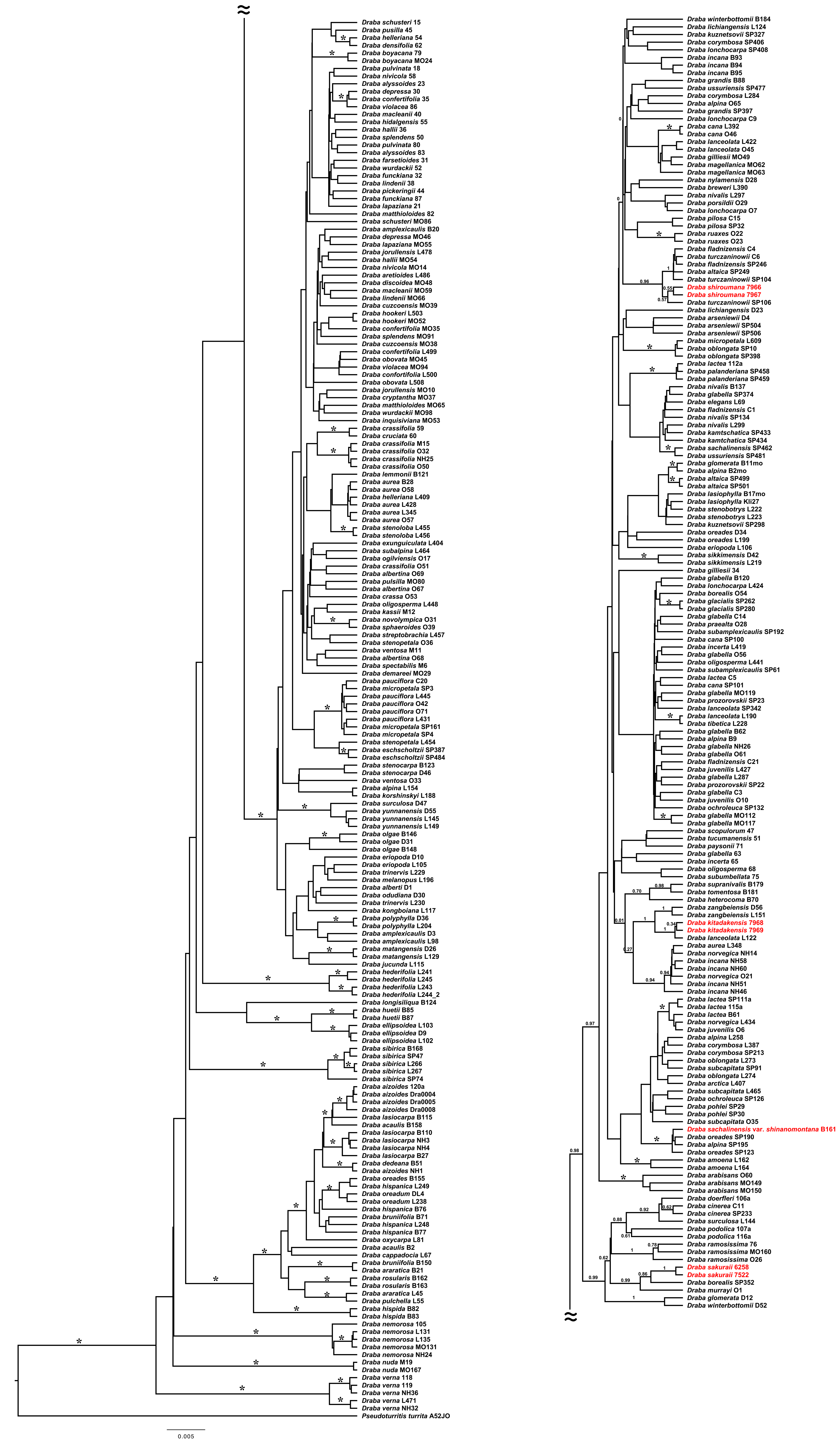


Fig. S2 The Bayesian tree based on ITS. The asterisk indicates strong posterior probability support (PP >= 95), and PP values for Japanese endemic taxa and their neighboring taxa are shown. The sample IDs are provided after the taxonomic name. Endemic taxa from central Honshu are indicated in colored text.

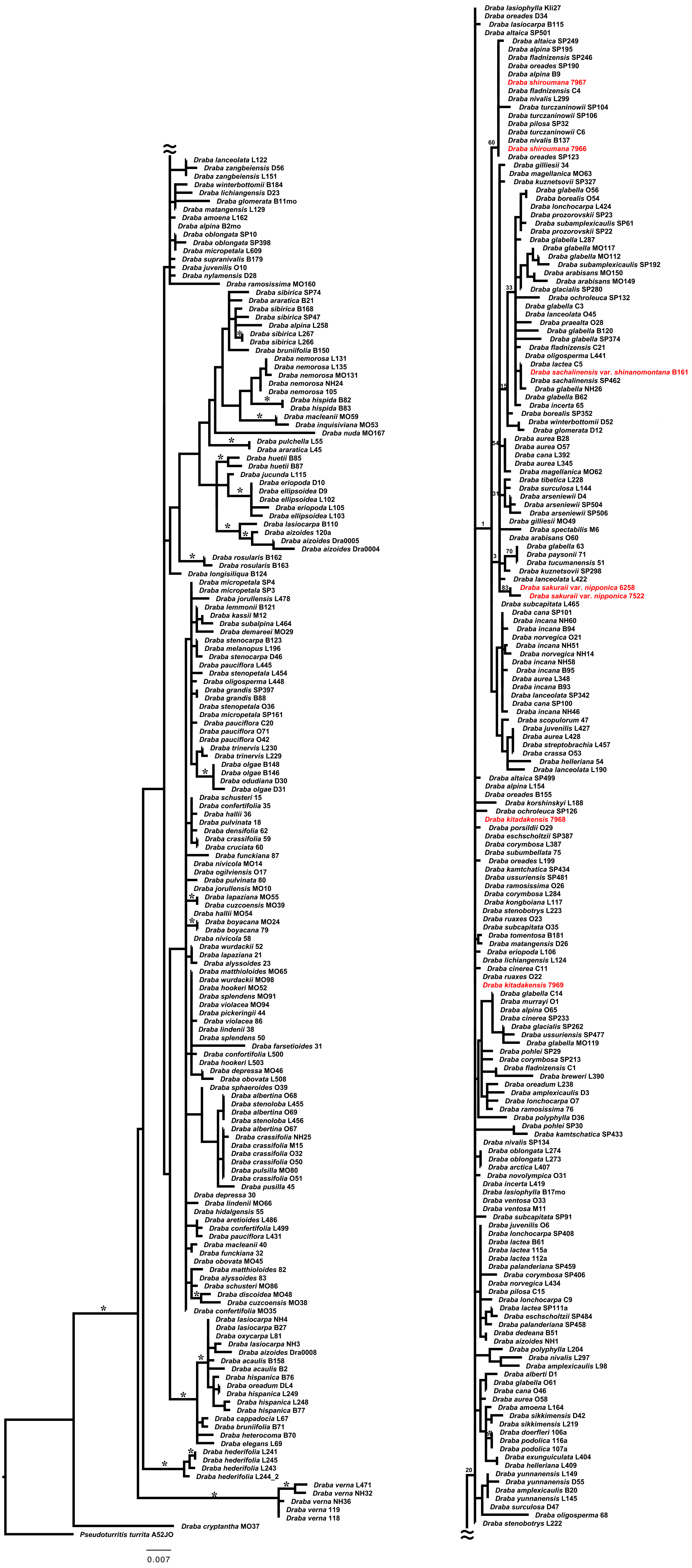


Fig. S3 The maximum likelihood tree based on *trnL-F*. The asterisk indicates strong bootstrap support (BS >= 80), and BS values for Japanese endemic taxa and their neighboring taxa are shown. The sample IDs are provided after the taxonomic name. Endemic taxa from central Honshu are indicated in colored text.

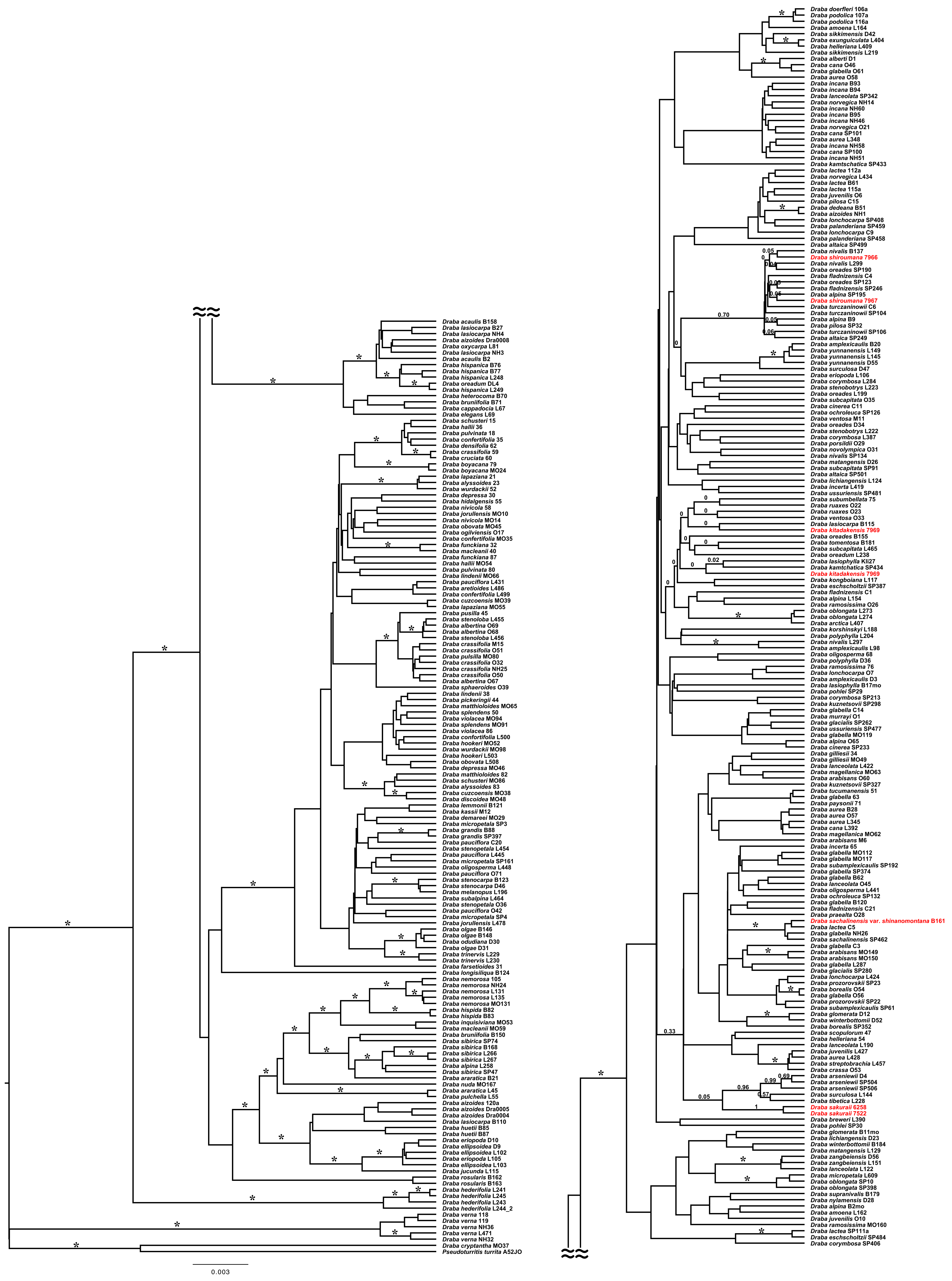

Fig. S4 The Bayesian tree based on *trnL-F*. The asterisk indicates strong posterior probability support (PP >= 95), and PP values for Japanese endemic taxa and their neighboring taxa are shown. The sample IDs are provided after the taxonomic name. Endemic taxa from central Honshu are indicated in colored text.

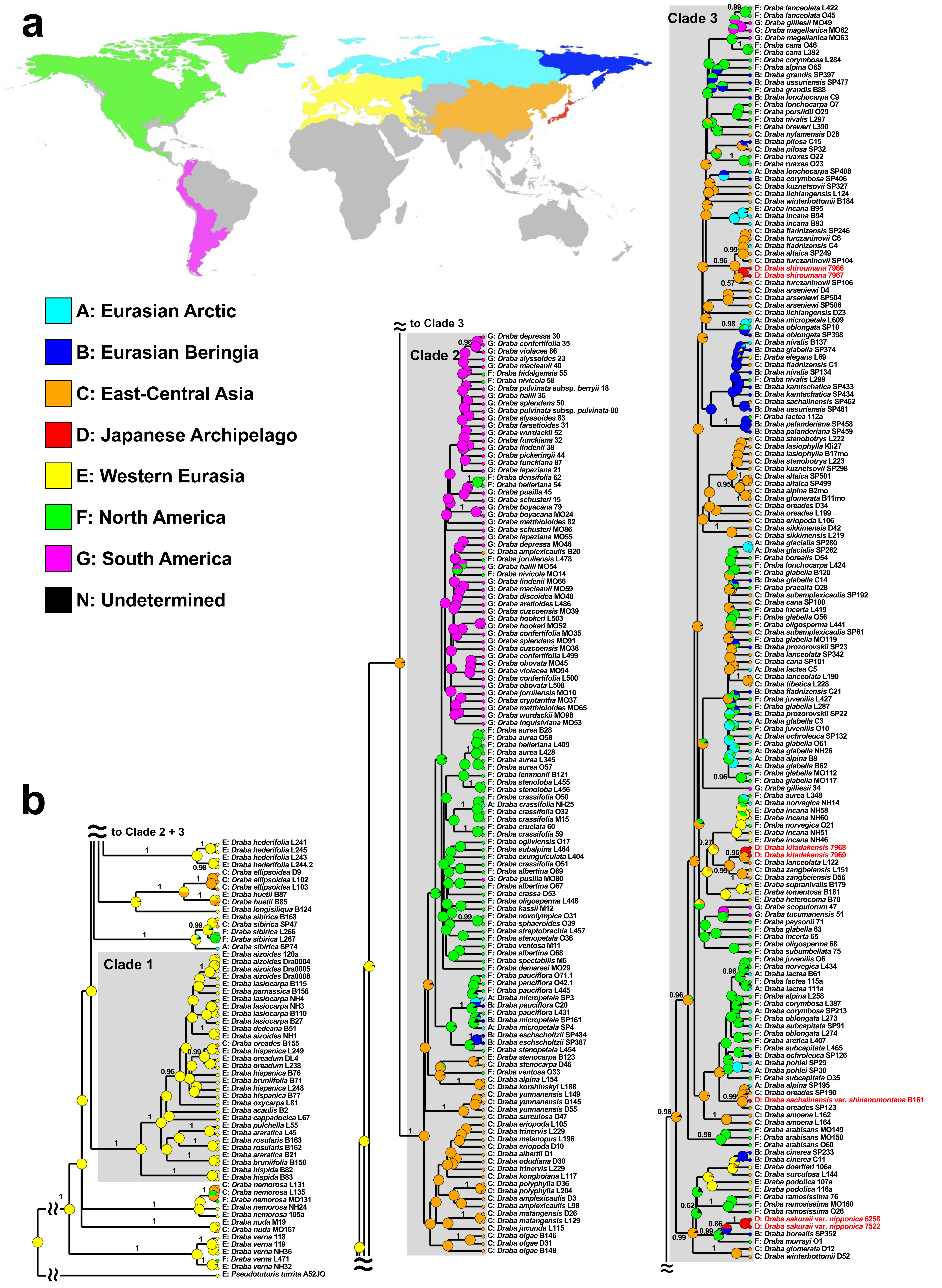


Fig. S5. Ancestral area reconstruction of the genus *Draba*. (a) Distribution map of the seven geographical regions used for reconstruction. (b) Bayesian tree based on ITS with estimated ancestral areas and likelihoods inferred using BioGeoBEARS, represented as pie charts at each node. Endemic taxa from central Honshu are indicated in colored text. Posterior probability support (PP ≥ 95) and PP values for neighboring taxa of the endemic *Draba* from central Honshu are indicated.
